# The SNARC effect: a preregistered study on the interaction of horizontal, vertical, and sagittal spatial–numerical associations

**DOI:** 10.1007/s00426-022-01721-8

**Published:** 2022-08-12

**Authors:** Sara Aleotti, Stefano Massaccesi, Konstantinos Priftis

**Affiliations:** 1 Department of General Psychology, University of Padua, Via Venezia 8, 35131 Padua, Italy; 2 Human Inspired Technology Center, University of Padua, Padua, Italy

## Abstract

Small numbers are processed faster through left-sided than right-sided responses, whereas large numbers are processed faster through right-sided than left-sided responses [i.e., the Spatial–Numerical Association of Response Codes (SNARC) effect]. This effect suggests that small numbers are mentally represented on the left side of space, whereas large numbers are mentally represented on the right side of space, along a mental number line. The SNARC effect has been widely investigated along the horizontal Cartesian axis (i.e., left–right). Aleotti et al. (Cognition 195:104111, 2020), however, have shown that the SNARC effect could also be observed along the vertical (i.e., small numbers-down side vs. large numbers-up side) and the sagittal axis (i.e., small numbers-near side vs. large numbers-far side). Here, we investigated whether the three Cartesian axes could interact to elicit the SNARC effect. Participants were asked to decide whether a centrally presented Arabic digit was odd or even. Responses were collected through an ad hoc-made response box on which the SNARC effect could be compatible for one, two, or three Cartesian axes. The results showed that the higher the number of SNARC-compatible Cartesian axes, the stronger the SNARC effect. We suggest that numbers are represented in a three-dimensional number space defined by interacting Cartesian axes.

## Numbers in the three-dimensional space

The mental representation of number magnitude is thought to be conveyed through a spatial format. The main experimental evidence of the relation between numbers and space is the Spatial–Numerical Association of Response Codes effect (SNARC; Dehaene et al., 1993). On a parity judgment task (i.e., decide whether a centrally presented number was odd or even), participants were faster in processing small numbers, with respect to large numbers, through left-sided responses. On the contrary, participants were faster in processing large numbers, with respect to small numbers, through right-sided responses (Dehaene et al., 1993). The SNARC effect has been considered as the most consistent evidence of a spatial–numerical association (SNA; for a comprehensive terminological approach, see Cipora et al., 2020; Shaki & Fischer, 2018) that resembles a horizontally aligned vector along which increasing number magnitude is represented from left to right (i.e., the mental number line: MNL; for a review, see Fischer & Shaki, 2015).

Although the MNL account has been highly influential, it is not the only one to address the issue of the mental representation of number magnitude (see Aleotti et al., 2020). In fact, two other accounts have been presented to explain the SNARC effect. The first one is the polarity correspondence account (Proctor & Cho, 2006). The second is the verbal working memory account (for a review, see Abrahamse et al., 2016).

The *polarity correspondence account* (Proctor & Cho, 2006) suggests that the SNARC effect (and other similar effects) is due to stimulus–response compatibility. For example, number magnitude could be coded through opposite polarities (i.e., small numbers: -; large numbers: +). Accordingly, lateralized responses could be coded by means of opposite polarities (i.e., left-sided responses: -; right-sided responses: +). When polarities are compatible (e.g., small numbers [-] processed through responses executed on the left side of space [-]), responses are faster than when polarities are incompatible (e.g., small numbers [-] processed through responses executed on the right side of space [ +]).

According to the *working memory account* (for a review, see Abrahamse et al., 2016), the SNARC effect would not reflect the structure and properties of a left-to-right oriented MNL (i.e., cardinal information) stored in long-term memory. Instead, the SNARC effect would be due to positional spatial codes (i.e., position markers) along left-to-right oriented series of items that are ordered in verbal working memory (i.e., ordinal information). By means of continuous electroencephalographic measures, Rasoulzadeh et al. (2021) have recently shown that position marking is mediated by horizontal spatial attention shifts to retrieve serial information in verbal working memory (see also Sahan et al., 2021).

Spatial cognition involves three dimensions (i.e., Cartesian axes: horizontal, vertical, and sagittal). Nevertheless, the main evidence on the mental association between numbers and space has been provided by investigating the SNARC effect on the horizontal axis (for a review, see Winter et al., 2015). In most of the SNARC paradigms, participants were asked to perform an arithmetic task (e.g., parity judgment) by means of horizontally arranged binary responses, which occurred on either the left or right side of space (Fischer & Shaki, 2014). Consequently, even though spatial representations are not restricted only to the horizontal axis, the investigation of the SNARC effect along other spatial dimensions (i.e., vertical and sagittal) has been limited. Recent studies, however, have shown that numbers might also be represented along the vertical (i.e., small numbers-down space vs. large numbers-up space) and sagittal (i.e., small numbers-near space vs. large numbers-far space) axes (for a review, see Winter et al., 2015).

Evidence for vertical spatial–numerical mapping was first reported in studies employing Random Number Generation (RNG; Grade et al., 2013; Hartmann et al., 2012; Loetscher et al., 2010; Winter & Matlock, 2013). In these studies, participants were asked to produce a random sequence of numbers, while performing downward or upward movements (e.g., head movements or body lifting). The results showed that participants generated smaller numbers after downward movements and larger numbers after upward movements.

The vertical SNARC effect has also been observed through eye-tracking paradigms (Schwarz & Keus, 2004; Experiment 2), while participants were asked to judge the parity of a centrally presented number. To perform the task, participants had to execute a saccade either towards a box placed on the upper side or towards a box placed on the lower side of a computer screen (e.g., even number-low side vs. odd number-up side). For smaller numbers, saccadic latencies were shorter when participants looked towards the lower box than towards the upper box. On the contrary, for larger numbers, saccadic latencies were shorter when participants looked towards the upper box than towards the lower box.

To date, only a few studies have investigated the vertical SNARC effect by means of bimanual number classification tasks (e.g., parity judgment task) through a vertical arrangement of the response buttons. Hartmann et al., (2014; Experiment 1) reported that smaller numbers were processed faster through down-sided responses, whereas larger numbers were processed faster through up-sided responses. On the contrary, Wiemers et al. (2017) did not replicate the results of Hartmann et al. Furthermore, Wiemers et al. reported evidence for an anatomical vertical SNARC effect. That is, participants responded faster to smaller numbers with their left hand, whereas they responded faster to larger numbers with their right hand, regardless of the arrangement of the response buttons (i.e., down vs. up). This finding suggested a dominance of hand-based mapping over the vertical mental representation of numbers.

Holmes and Lourenco (2012) also tested the vertical SNA on a parity judgment task, by means of two response boxes, which were vertically arranged on a touch-screen. The results yielded a vertical SNARC effect, but not when participants responded spontaneously. Indeed, the vertical SNARC effect was elicited only when the vertical dimension was primed by task instructions (e.g., to think about numbers as floors in a building). Finally, Sixtus et al. (2019) tested *"direction SNAs",* during *"implicit magnitude processing"* and *"implicit spatial directional processing"* (Cipora et al., 2020; Shaki & Fischer, 2018) along the horizontal and vertical axes. The results showed that large numbers were more strongly associated with upper space than small numbers.

A possible methodological limit is that in several studies on the vertical SNARC effect, through bimanual speeded responses, near/far response buttons instead of down/up response buttons have been employed (Gevers et al., 2006; Ito & Hatta, 2004; Shaki & Fischer, 2012; for the methodological issues, see Aleotti et al., 2020; Winter et al., 2015). Therefore, although these results were interpreted in terms of a vertical SNARC effect, actually the sagittal axis, instead of the vertical one, was tested.

Even fewer studies have focused on the sagittal SNA. Chen et al., (2015; Experiment 1) examined the association between the sagittal axis (i.e., near vs. far) and number representation. A 90° counterclockwise rotated computer keyboard was used (i.e., near button: Q key; far button: P key), while participants had to judge the parity of Arabic digits by pressing one of the near/far buttons. The results pointed towards a preferential mapping of small numbers in the near space and of large numbers in the far space, suggesting the existence of a sagittal SNARC effect. Finally, Lohmann et al. (2018) explored, through virtual reality, the modulation of the SNARC effect as a function of the distance of the numbers from the participants’ hands. The results showed that the SNARC effect was stronger when stimuli were presented near the hands or just outside reachable space.

To provide further evidence of the SNARC effect along all three Cartesian axes, Aleotti et al. (2020) investigated the three-dimensional SNARC effect by involving all Cartesian axes (i.e., horizontal, vertical, and sagittal), through the same sample of participants and by means of the same experimental paradigm. In separate sessions, one for each axis, participants were asked to perform a parity judgment task with response buttons, which were arranged horizontally, vertically, or sagittally. More precisely, responses were collected through an ad hoc-made response box composed of three orthogonal bars, one for each Cartesian axis (i.e., horizontal: left-sided/right-sided button; vertical: down-sided/up-sided button; sagittal: near-sided/far-sided button).

Aleotti et al. (2020) found three independent and equally strong SNARC effects, one for each Cartesian axis, supporting the concept of a three-dimensional mental number space. In this regard, smaller numbers were more associated with the left, down, and near sides of the mental space. On the contrary, larger numbers were more associated with the right, up, and far sides of the mental space (see also Hesse & Bremmer, 2017; Schwarz & Keus, 2004; Winter et al., 2015).

In most studies on the SNARC effect, the three Cartesian axes have been tested separately. Usually, the horizontal, vertical, and sagittal axes have been compared in different experimental blocks, in which the response buttons were arranged along a single Cartesian axis (e.g., Aleotti et al., 2020). Some evidence, however, has contributed to clarify the possible interaction of the Cartesian axes by combining the horizontal and vertical axes in diagonal response mappings (Gevers et al., 2006, Experiment 2; Hesse & Bremmer, 2017; Holmes & Lourenco, 2012).

Gevers et al., (2006; Experiment 2) used diagonal response mappings. In the left diagonal response mapping, there were a left-far key and a right-near key for executing responses (i.e., SNARC-incompatible on one axis, when compatible on the other). In the right diagonal response mapping, there were a left-near key and a right-far key for executing responses (i.e., SNARC-compatible on both axes). The results yielded a SNARC effect only when small numbers were processed with a left-near key, and large numbers were processed with a right-far key. Thus, the SNARC effect was elicited only when both the horizontal and sagittal axes were compatible. Note, however, that Gevers et al. termed the sagittal axis as vertical (see above).

Holmes and Lourenco (2012; Experiment 1B) also exploited diagonal response mappings, by means of a touch-screen assembly, on a parity judgment task. In compatible conditions, for small numbers, participants pressed the response box located on the left-down side of the screen, whereas for large numbers, they pressed the response box on the right-up side of the screen. Conversely, under incompatible conditions, for small numbers, participants pressed the response box located on the left-up side of the screen, whereas, for large numbers, they pressed the response box on the right-down side of the screen. The results showed that the SNARC effect was elicited under compatible conditions only. These results also highlighted that, when the horizontal and vertical axes were compatible with the SNARC effect, the participants' responses were faster than when either the horizontal or vertical axis was incompatible.

Hesse and Bremmer (2017) also compared the SNARC effect along both diagonal axes (left-down/right-up vs. left-up/right-down). Hesse and Bremmer asked participants to perform saccadic eye movements on a parity judgment task. The results showed that the SNARC effect emerged only when responses were towards the left-down position for small numbers and towards the right-up position for large numbers, confirming the findings of Holmes and Lourenco (2012).

Taken together, there is some evidence for the interaction of SNARC effects among the Cartesian axes through diagonal response mappings. In all previous studies, however, the diagonal response mappings derived from merging only two out of the three Cartesian axes (i.e., the horizontal and vertical axes or the horizontal and sagittal ones). Indeed, to date, no studies have investigated the SNARC effect by combining all three Cartesian axes (i.e., horizontal, vertical, and sagittal) simultaneously. Indeed, unless diagonal responses are used involving all the three axes simultaneously, it is impossible to reliably determine whether the SNARC effects, on each Cartesian axis (horizontal, vertical, and sagittal), could interact to achieve the combined SNARC effect.

The interaction of the three Cartesian axes is of paramount importance. Indeed, only through the interaction among the horizontal, vertical, and sagittal axis a point can be localized in space by means of the respective Cartesian coordinates. For example, a point that is in the far, upper, right space can be localized through the following Cartesian coordinates: *X*-axis = + 25, *Y*-axis = + 65, *Z*-axis =  + 75. Unless the Cartesian axes interact, no point can be localized in the three-dimensional space. The same is true for localizing any point in all the bidimensional spaces (horizontal-vertical, horizontal-sagittal, and vertical-sagittal). Thus, the interaction among the Cartesian axes is necessary in mathematics and in everyday life.

We aimed to test whether the horizontal, vertical, and sagittal SNAs could conceptually interact,^1^ by investigating the SNARC effect through diagonal response mappings, on a parity judgment task. According to the terminology coined by Cipora et al. (2020) and Shaki and Fischer (2018), by means of the SNARC effect, we assessed “direction SNAs”, during “implicit magnitude processing”, and “explicit spatial directional processing”.

**Fig. 1:**
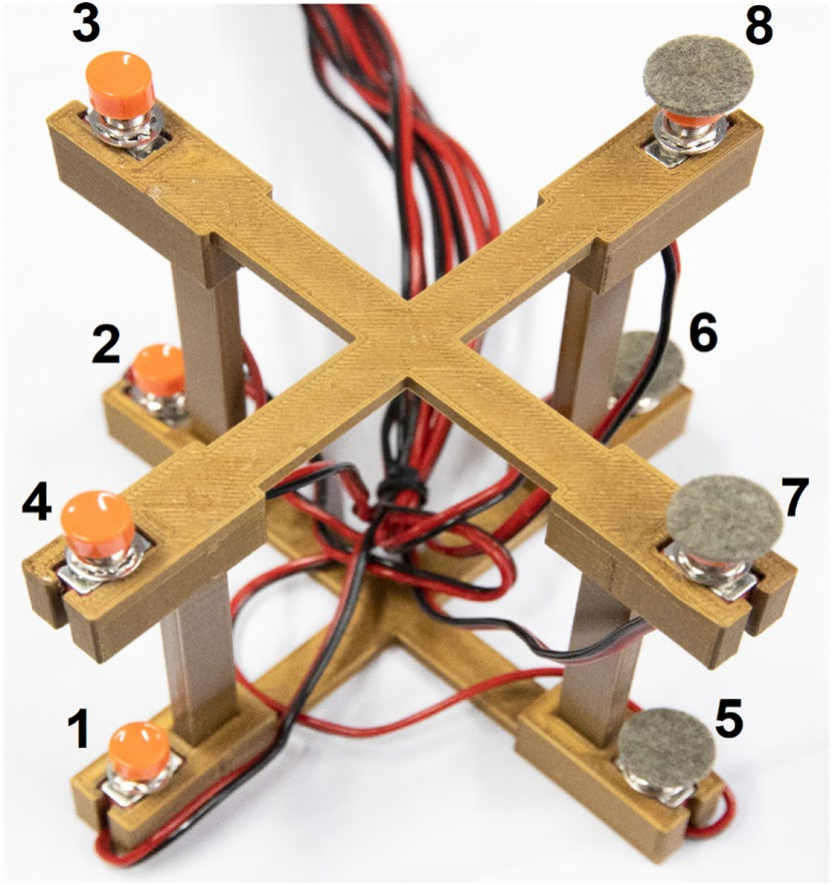
The ad hoc-made response box. The response box as seen from the side of the participant. The terms right/left, up/down, and near/far are referred to the viewpoint of the participant. Each button was placed in one of the eight spatial locations: 1 = left-down-near space; 2 = left-down-far space; 3 = left-up-far space; 4 = left-up-near space; 5 = right-down-near space; 6 = right-down-far space; 7 = right-up-near space; 8 = right-up-far space

**Fig. 2:**
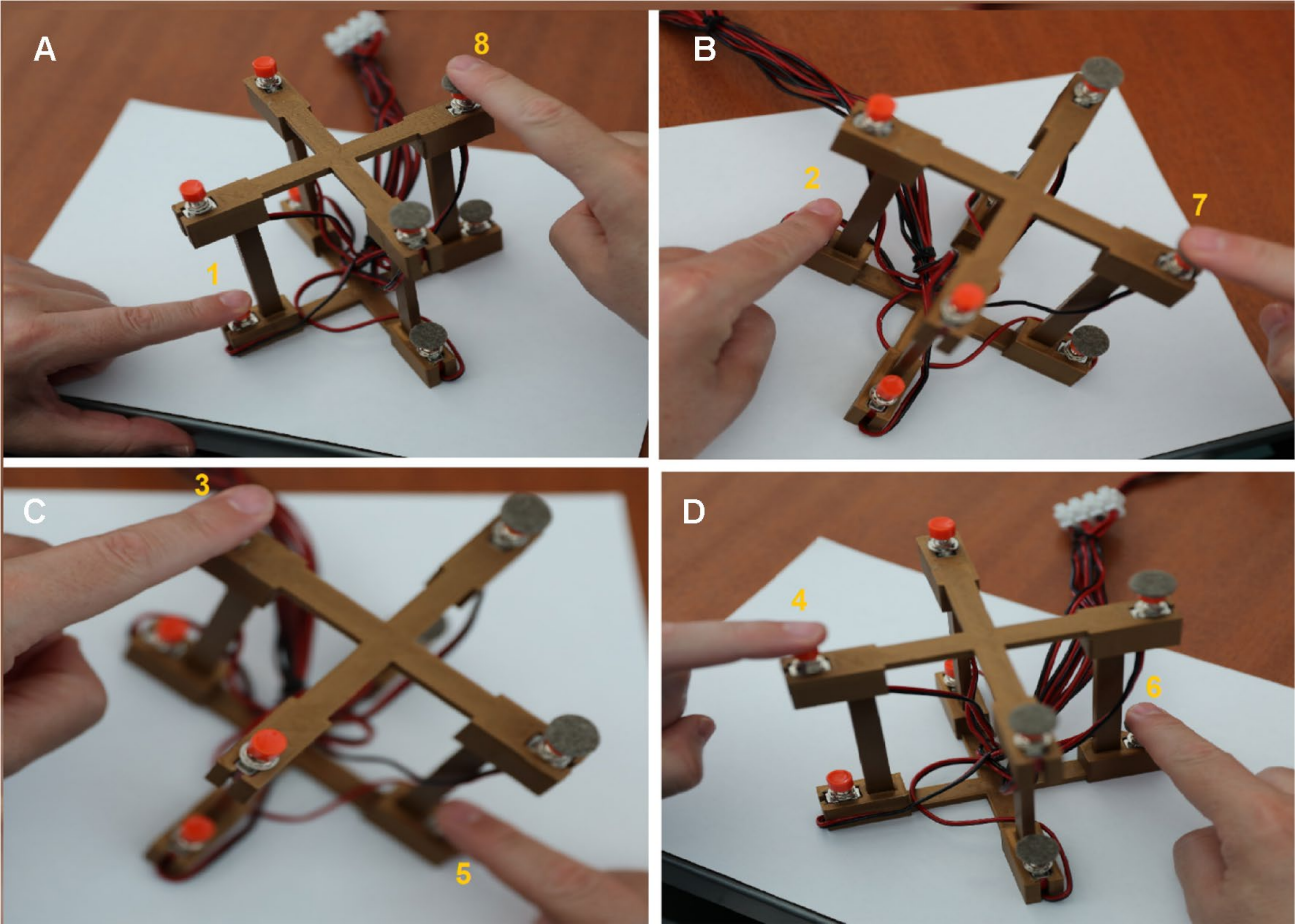
Response hand assignment for each diagonal response mapping. **A** Plane 1 (SNARC-compatible with all three Cartesian axes); **B** Plane 2 (SNARC-compatible on the horizontal and vertical axes, but not on the sagittal axis); **C** Plane 3 (SNARC-compatible only on the horizontal axis); **D** Plane 4 (SNARC-compatible on the horizontal and sagittal axes, but not on the vertical axis)

Our specific aim was to shed light on the contribution of each Cartesian axis to the SNARC effect. In this regard, we combined the three Cartesian axes to obtain four diagonal response mappings (i.e., Plane 1, Plane 2, Plane 3, and Plane 4; see Figs. 1 and 2). The diagonal response mappings resulted from the combination of each side of the horizontal axis (i.e., left and right), the vertical axis (i.e., down and up), and the sagittal axis (i.e., near and far). In our paradigm, number magnitude could be SNARC-compatible on one, two, or three Cartesian axes depending on the plane of reference (Table 1, Fig. 2). Therefore, by focusing on the presence and strength of the SNARC effect across planes, we investigated whether and which Cartesian axes interacted.

**Table 1:** Compatibility among axes and number magnitude

Axes	Plane 1		Plane 2		Plane 3		Plane 4	
	Left down near space	Right up far space	Left down far space	Right up near space	Left up far space	Right down near space	Left up near space	Right down far space
Small numbers								
Horizontal	+	−	+	−	+	−	+	−
Vertical	+	−	+	−	−	+	−	+
Sagittal	+	−	−	+	−	+	+	−
Large numbers								
Horizontal	−	+	−	+	−	+	−	+
Vertical	−	+	−	+	+	−	+	−
Sagittal	−	+	+	−	+	−	−	+

According to number magnitude, the Cartesian axes (i.e., horizontal, vertical, and sagittal) were compatible with the SNARC effect across the Planes (i.e., Plane 1, Plane 2, Plane 3, and Plane 4). “+” = SNARC-compatible; “−” = SNARC-incompatible. The SNARC effect was compatible with one (i.e., Plane 3), two (i.e., Plane 2 and Plane 4), or three (i.e., Plane 1) Cartesian axes

We formulated the following hypotheses:

i. If the horizontal and vertical axes conceptually interacted, then we would expect a significant SNARC effect elicited by Plane 1 and Plane 2, but no SNARC effect elicited by Plane 3 and Plane 4. The strength of the SNARC effect would not differ between Plane 1 and Plane 2. Alternatively, the SNARC effect might be present in all planes (i.e., Plane 1, Plane 2, Plane 3, and Plane 4). Nevertheless, if only the horizontal and vertical axes interacted, then, by combining Plane 1 with Plane 2 and Plane 3 with Plane 4, the strength of the SNARC effect given by the combination Plane 1-Plane 2 would be larger than the strength of the SNARC elicited by the combination Plane 3–Plane 4.
ii. If the horizontal and sagittal axes interacted, then we would expect a significant SNARC effect elicited by Plane 1 and Plane 4, but no SNARC effect elicited by Plane 2 and Plane 3. The strength of the SNARC effect would not differ between Plane 1 and Plane 4. The interaction between the horizontal and sagittal axes might exist even when the SNARC effect was elicited by all planes (i.e., Plane 1, Plane 2, Plane 3, and Plane 4). More precisely, if only the horizontal and sagittal axes interacted, then, by combining Plane 1 with Plane 4 and Plane 2 with Plane 3, the strength of the SNARC effect, given by the combined Plane 1-Plane 4, would be larger than the strength of the SNARC effect elicited by the combined Plane 2-Plane 3.
iii. If only the vertical and sagittal axes interacted, then we would expect a significant SNARC effect elicited only by Plane 1, but no SNARC effect elicited by Plane 2, Plane 3, and Plane 4. Nonetheless, the same pattern of results could also be observed when the three axes interacted (i.e., horizontal, vertical, and sagittal).
iv. If only one specific combination of the three Cartesian axes interacted, we would expect the SNARC effect along only that plane (i.e., Plane 1 or Plane 2 or Plane 3 or Plane 4).^2^

## Method

### Participants

Eighty-two Italian students (57 females; age: *M* = 22, SD = 2.89), from the University of Padua, voluntarily participated in the experiment. There was no compensation for participating in this study (i.e., money, credits, etc.). The participants were either right-handed (i.e., 78 participants) or left-handed (i.e., four participants). All participants had normal or corrected-to-normal visual acuity and were naïve about the purpose of the experiment. The number of participants was defined a priori, by means of the software G*POWER 3 (one-way repeated-measures ANOVA, four levels; Power = 0.99, *α* = 0.001, effect size = 0.25, *r* = 0.50; Faul et al., 2007). The study was approved by the local Ethics Committee (protocol number: 2892).

### Stimuli and apparatus

The following stimuli were used:

a) A white central cross (dimension: 1°);

b) White Arabic digits (dimension: 1.5°; small numbers: 2 and 3; large numbers: 7 and 8).

c) A mask (i.e., a white rectangle; dimensions: 15.5° × 10.2°).

All stimuli were presented in the center of a computer screen, against a black background (Cathodic Ray Tube monitor; dimension: 18″; colors: 32; refresh rate: 85 Hz; resolution: 1024 × 768 pixels; video card: INTEL(R) HD, graphics 530; RAM: 8 GB). The responses of the participants were collected by means of an ad hoc-made response box (Fig. 1). The buttons in the response box resembled the vertices of a virtual cube. On each vertex of the virtual cube, a button was placed, for a total of eight buttons.

The spatial position of each button resulted by combining all the three Cartesian axes (i.e., horizontal, vertical, and sagittal) with each other (i.e., left/right, down/up, and near/far). Therefore, four diagonal response mappings were established (i.e., Plane 1, Plane 2, Plane 3, and Plane 4; see Fig. 2). Each plane had two response buttons located at two opposite response box vertices. The distance between the buttons of each Plane (center to center) was 18.5 cm. Moreover, each Plane had one button with a felt pad (i.e., on the right extremity) and one smooth button (i.e., on the left extremity).

The experiment was programmed and administered to the participants by means of E-prime2 ProRC 8 (Psychology Software Tools, Inc., 2012). The experiment was carried out on an INTEL® Pentium® HP Compaq 6200 Pro SFF PC; operating system: Windows 7 Professional; CPU: G620, 2.60 GHz; RAM: 4.00 GB.

### Procedure

The participants sat in front of the computer screen in a dark and quiet room. Their head was placed on a chinrest at a distance of 57 cm from the screen, while their index fingers (i.e., left and right) were located on the respective response box buttons. For each plane separately, the experimenter first indicated the right index finger of the participant. Then, the participant received the following instruction: *“Please put this finger on this button*” (the experimenter indicated the button with the felt pad). Thereafter, the experimenter indicated the left index finger of the participant. Afterwards, the participant received the following instruction: *“Please put this finger on this button*” (the experimenter indicated the smooth button).

Participants performed a parity judgment task (i.e., they decided whether the Arabic digit displayed was odd or even) by pressing, as fast and accurately as possible, one of the two assigned response buttons. The experiment consisted of four sessions, one for each diagonal response mapping (Session 1: Plane 1; Session 2: Plane 2; Session 3: Plane 3; Session: 4 Plane 4). Half of the participants performed the sequence: Session 1 Session 2 Session 3 Session 4. The other half of the participants performed the opposite sequence: Session 4 Session 3 Session 2 Session 1. Moreover, all participants performed two different response button assignments in each session, which were counterbalanced between participants (Block 1: even number-smooth button vs. odd number-button with the felt pad; Block 2: even number-button with the felt pad vs. odd number-smooth button).

**Fig. 3:**
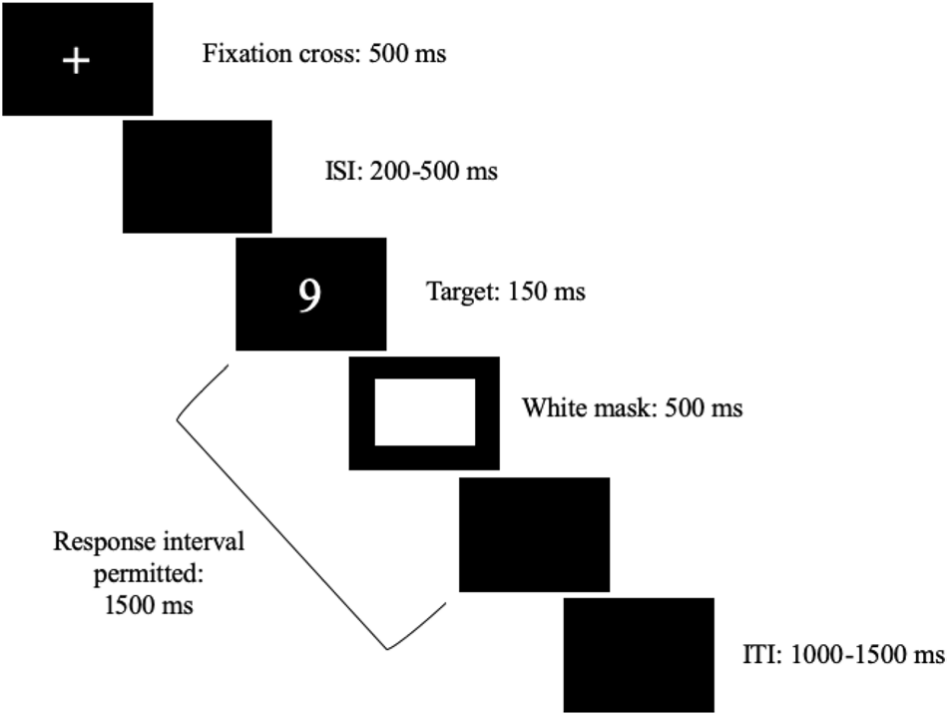
Trial sequence

Each trial started with the presentation of a central cross (500 ms; see Fig. 3) followed by a random inter-stimulus interval (ISI = 200–500 ms). Then, an Arabic digit appeared in the center of the screen for 150 ms. Thereafter, a white mask appeared for 500 ms. Afterwards, a black blank screen appeared for 850 ms. RTs could be recorded from the onset of the target stimulus until a response was executed over a maximum time interval of 1500 ms. Finally, a variable inter-trial interval (ITI: black blank screen; duration = 1000–1500 ms) elapsed before the next trial began. Overall, each participant performed 640 trials (160 trials for each session) and received eight practice trials (i.e., two for each digit), before each experimental block. The entire experiment lasted about 60'.

### Design

A within-participants experimental design was used. The independent variable was Plane (four levels: Plane 1, Plane 2, Plane 3, and Plane 4). The dependent variable was the dRT (RTs right hand–RTs left hand). More precisely, for each participant, for each Plane, and for each Arabic digit (i.e., 2, 3, 7, and 8), the difference between the RTs of the right hand and the RTs of the left hand was calculated.

We preregistered the present study on Open Science Framework, on September 24th, 2019: https://osf.io/x5dms. All data, analyses, and materials are available from https://osf.io/eusgq/

## Results

Frequentist analyses were performed with both IBM SPSS 24 and JASP Team (2018). Bayesian analyses were carried out by means of JASP. To perform Bayesian analyses, we used the default values of JASP. To interpret BF_10_, we considered the guidelines proposed by Wagenmakers et al. (2018).

Error rate (i.e., incorrect responses and omissions) was 4.16% of all trials. Three of the 82 participants were excluded from the statistical analyses, because their overall accuracy was less than 2.5 SD from the mean accuracy of all participants (*M* = 96%; SD = 0.04). Thus, all successive analyses were performed on the remaining 79 participants. Correct RTs, faster or slower than 2.5 SD from each condition’s mean, and for each participant, were excluded from the successive analyses (i.e., 2.31% of correct RTs discarded).

For each participant, a regression analysis procedure for repeated-measures designs was performed (Method 3; Lorch & Myers, 1990), separately for each plane (i.e., Plane 1, Plane 2, Plane 3, and Plane 4). The independent variable was the Arabic digits, whereas the dependent variable was the dRT. Note that the SNARC effect was qualified by a significant negative slope (Fias et al., 1996). Positive dRTs indicated faster left-sided than right-sided responses to small numbers. In contrast, negative dRTs indicated faster right-sided than left-sided responses to large numbers. Finally, the unstandardized beta regression coefficients of each Plane (Plane 1, Plane 2, Plane 3, and Plane 4), were subjected to a one-way repeated-measures analysis of variance (ANOVA).

**Fig. 4:**
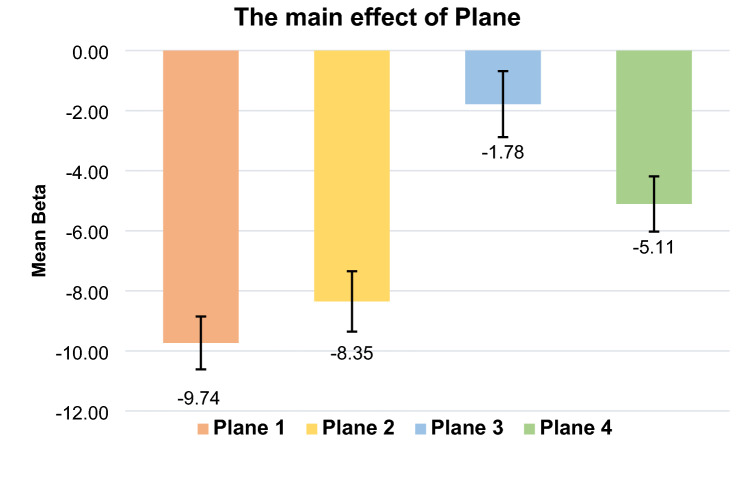
The main effect of plane on mean beta. Error bars show confidence intervals (95%) corrected for within-participants designs (O’Brien & Cousineau, 2014; single method)

The main effect of Plane was significant *F*(3, 234) = 13.15, *p* < 0.001, *η*^2^_p_ = 0.144 (Fig. 4). The Bayesian one-way repeated-measures ANOVA indicated that the observed data were 255,656 times more likely under H_1_ than under H_0_ (i.e., extreme evidence for H_1_; error % = 0.549).

To further investigate the presence of the SNARC effect on each Plane, the unstandardized beta regression coefficients of each participant were compared against zero, through a one-sample t test. The results showed that the regression slopes of Plane 1, Plane 2, and Plane 4 were significantly different from zero, showing the presence of the SNARC effect along these planes (Table 2). These results were also supported by Bayesian one-sample t tests, which also produced anecdotal evidence for H_0_ on Plane 3 (Table 3).

**Table 2:** Frequentist one-sample T tests

					95% CI for Mean Difference			95% CI for Cohen's d	
Betas	*t*	*df*	*p*	Mean difference	Lower	Upper	Cohen's *d*	Lower	Upper
Plane 1	− 9.376	78	2.012e−14	− 9.736	− 11.803	− 7.668	− 1.055	− 1.328	− 0.777
Plane 2	− 6.355	78	1.289e−8	− 8.353	− 10.969	− 5.736	− 0.715	− 0.961	− 0.466
Plane 3	− 1.821	78	0.072	− 1.785	− 3.735	0.166	− 0.205	− 0.427	0.019
Plane 4	− 4.274	78	5.379e−5	− 5.108	− 7.487	− 2.728	− 0.481	− 0.713	− 0.246

For each plane, the beta coefficients were compared against zero

**Table 3:** Bayesian one-sample *T* tests

Betas	BF_10_	Error %
Plane 1	3.552e+11	1.060e−17
Plane 2	914,639.792	6.857e−12
Plane 3	0.597	1.808e−6
Plane 4	354.030	2.358e−8

For each plane, the beta coefficients were compared against zero

Paired-samples t tests were carried out to compare the mean slope of Plane 1 with the mean slope of Plane 2 and with that of Plane 4. Only the comparison Plane 1–Plane 4 was significant (Table 4). The results of the Bayesian paired-samples t tests were consistent with those of the frequentist analyses. That is, the results revealed a very strong evidence for H_1_, only for the comparison Plane 1-Plane 4 (see Table 5).

**Table 4:** Frequentist paired-samples *T* tests

						95% CI for mean difference			95% CI for Cohen's d	
Betas	*t*	*df*	*p*	Mean difference	SE difference	Lower	Upper	Cohen's *d*	Lower	Upper
Plane 1 vs Plane 2	− 1.149	78	.254	− 1.383	1.204	− 3.780	1.014	− 0.129	− 0.350	.093
Plane 1 vs Plane 4	− 3.666	78	4.485e−4	− 4.628	1.263	− 7.141	− 2.114	− 0.412	− 0.641	− 0.181

Note. Beta coefficients were compared across planes

**Table 5:** Bayesian paired-samples *T* tests

Betas	BF_10_	Error %
Plane 1 vs. Plane 2	.233	8.803e−6
Plane 1 vs. Plane 4	50.342	1.147e−7

Beta coefficients were compared across planes

## Discussion

In the present study, our aim was to explore whether the SNARC effects along the Cartesian axes (i.e., horizontal, vertical, and sagittal) simultaneously interacted with each other. We used the SNARC effect to investigate for the presence of SNAs. According to the terminology coined by Cipora et al. (2020) and Shaki and Fischer (2018), we assessed “*direction SNAs*”*,* during “*implicit magnitude processing*” and “*explicit spatial directional processing*”. More precisely, we investigated the SNARC effect by means of diagonal response mappings (i.e., Plane 1, Plane 2, Plane 3, and Plane 4). We created response mappings by combining, with one another, all sides of the horizontal (i.e., left vs. right), vertical (i.e., down vs. up), and sagittal (i.e., near vs. far) axes. Therefore, based on the number magnitude conveyed, each plane could be SNARC-compatible for the combination of one (i.e., Plane 3), two (i.e., Plane 2 and Plane 4), or three (i.e., Plane 1) Cartesian axes (see Table 1).

The main effect of Plane was significant, indicating that the combined SNARC effect differed among planes. Indeed, the SNARC effect was found along Plane 1, Plane 2, and Plane 4 but not along Plane 3. Note that the response mappings for Plane 3 were SNARC-compatible on only one single Cartesian axis (i.e., horizontal). On the contrary, the response mappings for Planes 2 and 4 were SNARC-compatible for two Cartesian axes (i.e., horizontal-vertical and horizontal-sagittal, respectively). Finally, for Plane 1, the response mappings were SNARC-compatible along all three Cartesian axes.

These findings highlighted that, when at least two of three Cartesian axes were SNARC-compatible, the SNARC effects on two or three axes interacted with each other eliciting a combined SNARC effect. These results also suggested that one single Cartesian axis could not elicit the SNARC effect if, concurrently, the other two Cartesian axes were SNARC-incompatible (i.e., Plane 3). Thus, to reach a combined SNARC effect, responses should be SNARC-compatible on at least two Cartesian axes.

The comparison among the slopes of the axes (i.e., Plane 1 vs. Plane 2 and Plane 1 vs. Plane 4) showed a significant difference between the regression slopes of Plane 1 and Plane 4. On the contrary, no differences were found between the regression slopes of Plane 1 and Plane 2. Therefore, the SNARC effect elicited by the triple horizontal–vertical–sagittal interaction was stronger only when compared to the SNARC effect driven by the double horizontal–sagittal interaction. Finally, we showed that the horizontal, vertical, and sagittal SNARC effects simultaneously interacted eliciting a combined SNARC effect.

A different picture emerged, nonetheless, when effect sizes and Bayes factors were considered. Indeed, the analyses of the effect size revealed that the SNARC effect on Plane 1 was the strongest among all planes, followed by the effect size of Plane 2, and, then, by the effect size of Plane 4 (see Table 2; but see confidence intervals of Planes 1 and 2). The lowest effect size was reported for Plane 3. Therefore, it might be speculated that the higher the number of Cartesian axes compatible with the SNARC effect, the stronger their interaction. Also, the analysis of BFs supported this claim. Indeed, the highest BF was that of Plane 1, followed in order of magnitude by those of Planes 2, 4, and 3.

The findings of previous studies have suggested the existence of multiple SNARC effects. Numbers can be spatially arranged along mental representations along all Cartesian axes (i.e., horizontal, vertical, and sagittal; for a review, see Winter et al., 2015). Consequently, studies on the SNARC effect have shown that smaller numbers are processed faster when associated with the left-, down-, or near-sided response buttons. On the contrary, larger numbers are processed faster when associated with the right-, up-, or far-sided response buttons (Aleotti et al., 2020; Chen et al., 2015; Holmes & Lourenco, 2012). In most of the studies, multiple SNARC effects were explored by assessing the horizontal, vertical, or sagittal SNARC effects by means of double-choice responses arranged along each Cartesian axis separately.

Less evidence is available on the possible interaction of the SNARC effects among the Cartesian axes using diagonal response mappings (Gevers et al., 2006; Hesse & Bremmer, 2017; Holmes & Lourenco, 2012). Taking into account the number magnitude, the congruent diagonal response mapping resulted when both the horizontal and vertical axes were SNARC-compatible (i.e., left-down button vs. right-up button). On the contrary, incongruent diagonal response mapping resulted when only one Cartesian axis (e.g., the horizontal axis) was SNARC-compatible. In these studies, the SNARC effect was elicited only in the congruent diagonal response mapping, showing that when the horizontal and vertical axes were both SNARC-compatible, the responses were faster than when only the horizontal axis was SNARC-compatible. Hence, the horizontal and vertical SNARC effects interacted with each other enhancing the combined SNARC effect.

In the present study, we investigated whether the horizontal, vertical, and sagittal SNARC effects simultaneously interacted by combining all Cartesian axes in diagonal response mappings (i.e., Plane 1, Plane 2, Plane 3, Plane 4; see Table 1). The logic behind this study was similar to those of the previous ones, but the sagittal axis was also included. Therefore, in our study, diagonal response mappings could be SNARC-compatible for one, two, or three Cartesian axes.

Our results suggest that the SNARC effects among all Cartesian axes could interact. Whenever the diagonal response mapping was SNARC-compatible on two (i.e., Plane 2 and Plane 4) or on three (i.e., Plane 1) Cartesian axes, a significant, combined SNARC effect was elicited. This was not the case when only one Cartesian axis was SNARC-compatible (i.e., Plane 3). In this case, the interaction between SNARC-incompatible Cartesian axes (i.e., vertical and sagittal) could have significantly reduced the strength of the SNARC effect in Plane 3.

Our results suggest that the SNARC effects on each Cartesian axis (horizontal, vertical, and sagittal), although independent (Aleotti et al., 2020), could nonetheless interact to produce a combined SNARC effect on each Plane. This might be considered as evidence for three independent but interacting MNLs. Nevertheless, the MNL, for explaining the SNARC effect, has been recently challenged by alternative accounts (see also Aleotti et al., 2020): the polarity correspondence account (Proctor & Cho, 2006) and the working memory account (for a review, see Abrahamse et al., 2016).

According to the polarity correspondence account, the SNARC effect could be considered as an example of general stimulus–response compatibility. For instance, number magnitude could be coded through opposite-polarity signs (e.g., small numbers: −; large numbers: +). Lateralized responses could also be coded through opposite-polarity signs (e.g., left-sided responses: −; right-sided responses: +). When polarity signs are compatible (e.g., small numbers [−] processed through responses executed on the left side of space [−]), RTs are faster than when polarity signs are incompatible (e.g., small numbers [−] processed through responses executed on the right side of space [+]).

According to the polarity account, one would have expected to observe an equally strong SNARC effect, on each of the four Planes. Indeed, polarity correspondence remained always the same on the four Planes. In contrast, we observed that the higher the number of SNARC-compatible axes on each Plane, the stronger the SNARC effect. Therefore, our results support the interaction of the horizontal, vertical, and sagittal MNLs in eliciting the SNARC effect, rather than the polarity correspondence account.

The working memory account (for a review, see Abrahamse et al., 2016) suggests that the SNARC effect does not reflect the activation of a left-to-right oriented MNL coding cardinal information. Instead, the SNARC effect would be due to positional spatial codes along left-to-right oriented series of items in verbal working memory, in which ordinal information is represented (Rasoulzadeh et al., 2021; Sahan et al., 2021). Therefore, the verbal working memory account could elegantly explain the SNARC effect observed along the horizontal axis. Nevertheless, the working memory account could not explain why the SNARC effect disappears (Plane 3) or becomes stronger (e.g., Plane 1) as a function of the overall number of SNARC-compatible versus SNARC-incompatible axes. Indeed, it could be presumed that the same verbal working memory resources are required on each of the four planes. If this had been the case, we would have yielded four equally strong SNARC effects, one for each Plane. By contrast, we found significant differences among the Planes according to the number of the SNARC-compatible versus SNARC-incompatible axes. Thus, the verbal working memory account should be expanded further to fully explain our findings.

One might wonder where the interaction of multiple SNARC effects occurs. The functional locus of the combined SNARC effects could be ascribed, on the one hand, to the spatial layout of the mental representation of number magnitude. If so, it would be possible to obtain the combined SNARC effects even without using lateralized response buttons (in this regard, see https://osf.io/w95tr). On the other hand, combined SNARC effects might originate at the level of response selection.

Basso Moro et al. (2018) aimed to disentangle these opposite views by measuring, in the same experiment, the modulation of the SNARC effect at both the semantic representational stage and the response-selection processing stage. To this purpose, the magnitude comparison task and stimulus-mapping switching task were combined in a unique paradigm. The findings of Basso Moro et al. pointed to a combination of causes, both semantic and response-selection related that concurrently determined the SNARC effect. These findings suggest that horizontal SNA could be observed in either of two cognitive processing stages (i.e., semantic processing and response selection).

Future studies would further investigate whether the interactions among the horizontal, vertical, and sagittal SNARC effects have their origin at the semantic or at the response-selection stage. The existence of the interaction among the three SNARC effects (i.e., horizontal, vertical, and sagittal) might have relevance for clarifying which Cartesian dimensions have a stronger influence on number processing.

In conclusion, our findings suggest that the three Cartesian axes (i.e., horizontal, vertical, and sagittal) can interact during number-magnitude processing. The higher the number of compatible Cartesian axes, the stronger the SNARC effect. This might be considered as evidence of a shared three-dimensional mental number space in which number magnitude might be represented either along one single Cartesian axis (i.e., three independent MNLs; Aleotti et al., 2020) or along diagonally combined Cartesian axes. We suggest that numbers are represented in a three-dimensional number space defined by interacting Cartesian axes. This interaction might have originally guided the invention of Cartesian coordinates. Our findings might further inform current theories on numerical cognition and lead to useful applications on devices displaying numbers in space (e.g., car dashboards, keyboards, gear levers, etc.). In follow-up studies, it would be very informative to compare the performance of samples with different ages, different mathematical abilities, different spatial abilities, and different cultures.

## Data Availability

We preregistered the present study on Open Science Framework on September 24th, 2019: https://osf.io/x5dms All data, analyses, and materials are available from https://osf.io/eusgq/.

## Footnotes

1 Note that the term “*interaction”* is conceptual. In particular, here, it refers to the *“conceptual interaction”* of the Cartesian axes on each plane. Therefore, in this context, the term “*interaction”* must not be interpreted as a statistical interaction among factors, given that we did not manipulate orthogonally the compatibility of each axis (i.e., horizontal, vertical, or sagittal) with the SNARC effect. Instead, with the term *“conceptual interaction”*, we mean that the higher the number of compatible axes in each plane, the stronger the SNARC effect. The conceptual meaning of axes interaction has been used also through the Discussion section.

2 With respect to the preregistered version of the present study, a part of this hypothesis has been omitted, because of a needless, overlapping comparison already present in the other hypotheses.
